# Exposure to the Dioxin-like Pollutant PCB 126 Afflicts Coronary Endothelial Cells via Increasing 4-Hydroxy-2 Nonenal: A Role for Aldehyde Dehydrogenase 2

**DOI:** 10.3390/toxics10060328

**Published:** 2022-06-16

**Authors:** Bipradas Roy, Zhao Yang, Guodong Pan, Katherine Roth, Manisha Agarwal, Rahul Sharma, Michael C. Petriello, Suresh Selvaraj Palaniyandi

**Affiliations:** 1 Division of Hypertension and Vascular Research, Department of Internal Medicine, Henry Ford Health System, Detroit, MI 48202, USA; biroy@med.wayne.edu (B.R.); gpan1@hfhs.org (G.P.); 2 Department of Physiology, Wayne State University, Detroit, MI 48202, USA; 3 Institute of Environmental Health Sciences, Wayne State University, Detroit, MI 48202, USA; zhaoyang@med.wayne.edu (Z.Y.); katherine.roth3@wayne.edu (K.R.); sharma.rahul@wayne.edu (R.S.); 4 Department of Pharmacology, School of Medicine, Wayne State University, Detroit, MI 48202, USA; manisha.agarwal@wayne.edu

**Keywords:** angiogenesis, 3, 3′, 4, 4′, 5-pentachlorobiphenyl (PCB 126), environmental pollutants, coronary endothelial cells, aldehyde dehydrogenase 2, 4-hydroxy-2-nonenal

## Abstract

Exposure to environmental pollutants, including dioxin-like polychlorinated biphenyls (PCBs), play an important role in vascular inflammation and cardiometabolic diseases (CMDs) by inducing oxidative stress. Earlier, we demonstrated that oxidative stress-mediated lipid peroxidation derived 4-hydroxy-2-nonenal (4HNE) contributes to CMDs by decreasing the angiogenesis of coronary endothelial cells (CECs). By detoxifying 4HNE, aldehyde dehydrogenase 2 (ALDH2), a mitochondrial enzyme, enhances CEC angiogenesis. Therefore, we hypothesize that ALDH2 activation attenuates a PCB 126-mediated 4HNE-induced decrease in CEC angiogenesis. To test our hypothesis, we treated cultured mouse CECs with 4.4 µM PCB 126 and performed spheroid and aortic ring sprouting assays, the ALDH2 activity assay, and Western blotting for the 4HNE adduct levels and real-time qPCR to determine the expression levels of *Cyp1b1* and oxidative stress-related genes. PCB 126 increased the gene expression and 4HNE adduct levels, whereas it decreased the ALDH2 activity and angiogenesis significantly in MCECs. However, pretreatment with 2.5 µM disulfiram (DSF), an ALDH2 inhibitor, or 10 µM Alda 1, an ALDH2 activator, before the PCB 126 challenge exacerbated and rescued the PCB 126-mediated decrease in coronary angiogenesis by modulating the 4HNE adduct levels respectively. Finally, we conclude that ALDH2 can be a therapeutic target to alleviate environmental pollutant-induced CMDs.

## 1. Introduction

Angiogenesis is the sprouting of new blood vessels from preexisting vessels. It is one of the most important physiological properties of endothelial cells (ECs) of the vascular tissue. Angiogenesis is essential in both physiological and pathological conditions, including embryonic development, organ perfusion, wound healing, tissue regeneration, and tumor growth [1]. Coronary angiogenesis is critical to maintain proper cardiac perfusion for regulating cardiac function, metabolism, and tissue regeneration. Decreased coronary angiogenesis leads to cardiometabolic diseases (CMDs), including cardiomyopathy, heart failure with preserved ejection fraction (HFpEF), and myocardial ischemia–reperfusion injury (IRI) [2]. The regulation of angiogenesis depends on a dynamic balance between proangiogenic and antiangiogenic processes [1]. Dysregulation of the balance between proangiogenic and antiangiogenic factors leads to either excess or insufficient angiogenesis.

Environmental pollutants play an important role in several pathological conditions, including cardiovascular diseases, pulmonary diseases, renal disease, and cancer. For instance, a study on 22 people with type 2 diabetes showed that exposure to fine airborne particulate matter (PM_2.5_) caused immediate endothelial dysfunction that was characterized by a reduced flow-mediated dilatation and small-artery elasticity index [3]. In addition to airborne pollutants, persistent organic pollutants, especially halogenated pollutants, have been implicated in cardiovascular risks. Dioxins and dioxin-like chemicals are a group of structurally related persistent organic pollutants that are largely generated by humans through industrial processes, including incineration, the production of herbicides and pesticides, and the use of fertilizers. Dioxins are highly toxic and have been found to work through a common mechanism of action that is mediated through activation of the Aryl hydrocarbon receptor (*AhR*). One class of dioxin-like pollutants, known as dioxin-like polychlorinated biphenyls (PCBs), including PCB 126, PCB 77, and PCB 169, have been manufactured as mixtures and utilized in a variety of commercial products, including additives to sealants and paints and in oils used in industrial processes for their insulation and fire-retardant properties. Dioxin-like polychlorinated biphenyl (PCB) 126 is one of the potent dioxin-like PCBs and a well-known environmental pollutant that is implicated in causing severe toxicity due to its bioaccumulation [4]. PCBs were eventually banned in the United States in the late 1970s and then worldwide in 2001 after the Stockholm Convention. Prior to this ban, multiple occupational and accidental exposures occurred, but now, the primary source of exposure to PCBs is through contaminated food, including high-fat foods such as meat, fish, and dairy products. Since PCBs are now ubiquitous contaminants in the global food chain, even humans in the general population have a body burden of PCBs and other dioxin-like pollutants. A cross-sectional analysis of 1844 men in their 50s and free of cardiovascular disease showed that dietary exposure to PCBs was associated with a higher prevalence of coronary calcium levels and intense subclinical coronary atherosclerosis [5]. Another population-based prospective investigation of the vasculature in Uppsala seniors (PIVUS) with 1016 participants showed an increase in atherosclerotic plaque formation in the carotid artery, along with an elevation of the serum PCBs levels independent of the cardiovascular risk factors, including lipids [6]. A separate population-based study examining the PIVUS cohort demonstrated that the prevalence of left ventricular hypertrophy is positively correlated with the serum levels of PCBs, including PCB 118, 126, 169, and 156 [7]. Another prospective population-based study that recruited 32,952 women and 36,546 men free from cancer, cardiovascular disease, and diabetes at the baseline showed that dietary exposure to PCBs was associated with an increased risk of HF in both women and men [8]. Several preclinical studies determined the role of PCBs in different pathophysiological conditions. For instance, an in vivo study on rodents showed increased body weights, impaired insulin sensitivity; reduced adipose tissue deposition; and elevated serum triglycerides, cholesterol, and insulin levels, along with increased free radical generation and altered protein levels related to oxidative stress in the islets of Langerhans upon PCB 126 exposure [9]. Another study by the same research group showed that the intranasal administration of PCB 126 exerts an innate immune deficiency that was characterized by the impairment of adhesion receptors on blood leukocytes and reduced blood neutrophil locomotion and G protein-coupled receptor (GPCR)-mediated activation of an oxidative burst in rats [10]. The administration of PCB 126 in mice through oral gavage increased the plasma levels of the inflammatory markers of the peripheral vasculature, including ICAM-1, plasminogen activator inhibitor-1, and proatherogenic trimethylamine-N-oxide [11]. Another in vitro study showed that a treatment with 0.03 nM PCB 126 significantly increased the expression of vascular inflammatory mediators, including interleukin (IL)-6, C-reactive protein (CRP), intercellular adhesion molecule-1 (ICAM-1), vascular cell adhesion molecule-1 (VCAM-1), and IL-1α/β, in the cultured human umbilical vein endothelial cell (HUVEC) lines [12]. Low-density lipoprotein receptor knockout mice showed increased levels of plasma proinflammatory cytokines, increased circulating biomarkers of CVD, altered platelet and red blood cell counts, an increased accumulation of hepatic fatty acids, and accelerated atherosclerotic lesion formation in the aortic root after 10 weeks of PCB 126 exposure [13]. In addition to proinflammatory, hypertrophic, and pro-atherosclerotic effects, PCBs have antiangiogenic effects as well. For instance, PCB treatment in HUVECs significantly decreased angiogenesis compared with the controls, as evident from an in vitro tube formation assay on Matrigel, as well as an aortic ring assay using mouse aorta [14].

Previously, we demonstrated that PCB 126 has a role in endothelial cell dysfunction, oxidative stress, and accelerated atherosclerosis [13], but its impacts on coronary angiogenesis have not been studied yet. In our cell culture studies using mouse coronary endothelial cells (MCECs), we demonstrated that a direct exogenous treatment of 4-hydroxy-2-nonenal (4HNE), a secondary metabolite of oxidative stress that is generated upon lipid peroxidation, reduces angiogenesis [1]. This antiangiogenic effect of 4HNE was aggravated by inhibiting aldehyde dehydrogenase 2 (ALDH2), a mitochondrial enzyme that metabolizes 4HNE, by pharmacologically inhibiting ALDH2 activity with disulfiram (DSF) [1]. In animal studies, we also found reduced coronary angiogenesis in mice with low intrinsic ALDH2 activity due to diabetic stress or a single-point mutation (E487K) in ALDH2, termed ALDH2*2 [15]. When we subjected the ALDH2*2 mutant diabetic mouse hearts to ischemia–reperfusion injury (IRI), we found the augmented apoptosis of CD31+ coronary endothelial cells, along with increased 4HNE adduct formation, compared to wild-type diabetic mouse hearts that underwent similar IRI [15].

Roughly ~30% of East Asians, i.e., ~400 million people, carry the ALDH2*2 mutation, leading to an increased incidence of cardiovascular diseases, including myocardial infarction, coronary spasm, diabetic cardiac complications, and heart failure [16]. Several East Asian countries have high environmental pollution, and the people in those countries experience PCB-induced health hazards. A recent study from China reported that ALDH2*2 mutant patients with metabolic disorders such as diabetes mellitus have a higher incidence of HF with preserved ejection fraction (HFpEF), a disease originating from coronary endothelial dysfunction [17].

Thus, understanding the mechanism of PCB 126-induced coronary endothelial cell damage can be beneficial in preventing several critical CMDs, including HFpEF. In this study, we specifically plan to determine if PCB 126-induced coronary endothelial cell damage is mediated via 4HNE and if modulating ALDH2 activity plays a role in this process.

## 2. Materials and Methods

### 2.1. Experimental Animal

Aortas from six-month-old *C57BL/6* mice were used for this study. Mice were bred and maintained in the animal care facility at Henry Ford Health System. Mice were humanely euthanized, and aortas were isolated for the aortic ring assay. The animal protocols were approved by the Wayne State University Institutional Animal Care and Use Committee, which conforms to NIH standards.

### 2.2. Cell Culture

The mouse coronary EC (MCEC) line was obtained from Cedarlane (#CLU510) and grown as we already described [1]. We used MCECs after the fourth passage, and they were grown in new DMEM supplemented with 0.2% FBS (low serum) and 1% P/S. After 24 h of low-serum treatment, the cells were used to perform the spheroid assay and subjected to the treatment protocols, as described below.

### 2.3. Treatment Protocols

**Figure 1:**
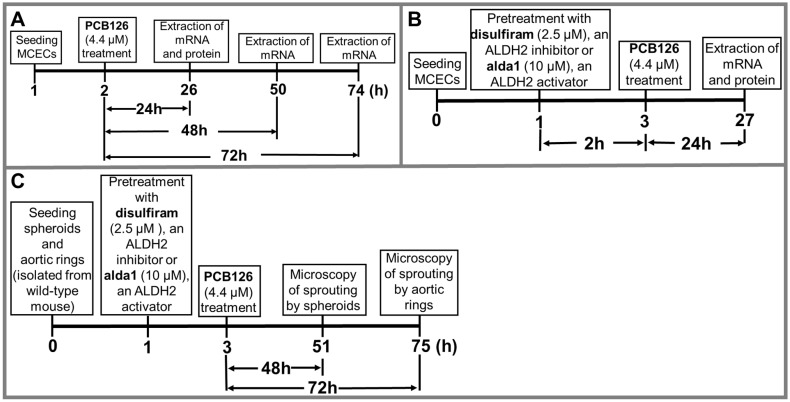
Treatment protocols.

Protocol 1: We treated MCECs with 4.4 µM of PCB 126 (AccuStandard Inc., New Haven, CT, USA) or the vehicle (dimethyl sulfoxide (DMSO) (#sc 358801)) for 24–72 h, followed by the extraction of mRNA and the protein to perform real-time polymerase chain reaction (RT-PCR), Western blotting (WB), and the ALDH2 activity assay, respectively (Figure 1A). An initial dose response study using 0, 0.44 µM, and 4.4 µM PCB 126 was used to determine an optimal dose for AhR activation.

Protocol 2: We treated MCECs with the vehicle (DMSO) or disulfiram (DSF) (#sc-205654A) (2.5 μM), an ALDH2 inhibitor, or Alda 1 (#A5823, Acme Bioscience) (10 µM), an ALDH2 activator, for 2 h before challenging with PCB 126 (4.4 µM) for 24 h. Finally, we extracted mRNA and proteins for real-time PCR (RT-qPCR) or Western blot analysis (Figure 1B).

Protocol 3: To study the role of PCB 126, along with the modulation of ALDH2 activity in coronary angiogenesis, we treated spheroids and aortic rings with 2.5 µM disulfiram (DSF), an ALDH2 inhibitor, or 10 µM Alda 1, an ALDH2 activator, for 2 h, followed by treatment with 4.4 µM PCB 126 for 48 h and 72 h, respectively. We performed microscopy for sprout growth by the spheroids and aortic rings after 48 and 72 h of PCB 126 treatment, respectively (Figure 1C).

### 2.4. Spheroid Assay

For making spheroids (~400 cells/spheroid), we diluted them in 4 mL of DMEM supplemented with 10% FBS, 1% P/S, and 20% Methocel (#M7027-250G, SIGMA). To form spheroids, we incubated the plate upside-down in a humidified incubator set at 37 °C with a continuous supply of 5% CO_2_ for 24 h. We then transferred the spheroid suspension in a 15-mL conical tube and centrifuged it at 200× *g* for 5 min. We aspirated the supernatant and added 2 mL ice-cold Methocel containing 20% FBS. Separately we prepared a 4 mL collagen stock solution by adding 1 mg/mL collagen (#32160405, MilliPORE SIGMA) in ice-cold DMEM supplemented with 10% FBS, 1% P/S, and 30 ng/mL VEGF (#493-MV, R&D Systems). We mixed ice-cold Methocel containing the spheroids with collagen stock solution and subsequently pipetted 250 μL of collagen stock containing the spheroids in each well of a 24-well cell culture plate. We incubated the plates containing the spheroids in a humidified incubator at 37 °C with a continuous supply of 5% CO_2_ for 1 h to solidify the collagen bed due to polymerization. We added 250 μL of DMEM supplemented with 10% FBS, 1% P/S, and 30 ng/mL VEGF in each well of the 24-well plate containing the collagen bed with spheroids embedded in it and subsequently incubated in a humidified incubator at 37 °C with a continuous supply of 5% CO_2_ for 1 h. Then, we treated the spheroids according to the protocol mentioned in Figure 1C. We captured images of sprout growth from the spheroids embedded in the collagen matrix using a 10× phase–contrast microscope. To determine the angiogenesis, we counted the number of nodes in sprouted spheroids under a high-power field (HPF) using ImageJ software.

### 2.5. Aortic Ring Assay

We performed the aortic ring assay as described previously [18]. Briefly, we isolated the aorta and washed with sterilized PBS to remove the residual blood. We immediately transferred the cleaned aorta to a 100-mm plate containing fresh Opti-MEM (#51985-034, ThermoFisher Scientific). Using a sharp and sterile scalpel, we dissected the aorta into small ring-like fragments with an approximate length of 1 mm. From a single aorta, we made 28–34 aortic rings. Using a sterile sharp-tipped tweezer, we transferred the aortic rings to a new 60-mm/6-well plate containing DMEM supplemented with 0.2% FBS and 1% P/S. We pipetted 200 μL of the collagen stock into each well of a 24-well plate and subsequently planted three aortic rings in each well on the top of the collagen bed using a sharp-tipped sterile tweezer. We planted the aortic rings on the collagen bed so that the luminal axis was perpendicular to the bottom of the well. Then, we incubated the plate in a humidified incubator set at 37 °C with a continuous supply of 5% CO_2_ for 1 h to solidify the collagen bed due to polymerization. We added 100 μL of collagen stock to each well on top of the aortic rings to embed them into the collagen bed. Then, we treated the aortic rings according to the protocol we discussed elsewhere in the manuscript (Figure 1C). We captured images of sprout growth from the aortic rings embedded in the collagen matrix using a 10× phase–contrast microscope. We measured the relative sprouting area (%) from each aortic ring under HPF to determine the angiogenesis using ImageJ software.

### 2.6. Real-Time qPCR

The total RNAs from MCECs purified by Trizol reagent (Invitrogen) were reverse-transcribed to cDNA for quantifying with an Applied Biosystems QuantStudio 6 Flex RT-*q*PCR System using SYBR Green (Applied Biosystems). Samples were analyzed as duplicates, and the expression levels were calculated using the ΔΔCt method. The PCR primers are described in Table 1. The housekeeping gene, Beta-actin (*Actb*), is listed at the bottom of the table. The PCB 126 treatment did not significantly impact the *Actb* expression.

### 2.7. ALDH2 Activity Assay

The ALDH2 activity was measured according to the protocol described elsewhere [18]. Briefly, MCECs were grown on 100-mm plates and treated according to the protocol in Figure 1B, and cellular protein was extracted, and 100 μg of total cellular protein from each sample was used for this assay. Freshly made 50 mM sodium pyrophosphate (#221368-500G, SIGMA) solution as a buffer, 2.5 mM NAD+ (#N3014-5G, SIGMA) solution as a cofactor, and 10 mM acetaldehyde (#402788-100ML, SIGMA) as a substrate were used. The enzymatic activity of ALDH2 from cell lysate was determined spectrophotometrically using the reductive reaction of NAD+ to NADH at a λ340-nm wavelength at 37 °C.

### 2.8. Western Immunoblotting

4HNE protein adducts and ALDH2 protein levels were evaluated using the WB assay, as we described earlier [18]. In brief, after treatment, the cellular protein was extracted from cultured MCECs using a tissue protein extraction reagent (ThermoFisher Scientific) containing protease/phosphatase inhibitors. Specific protein bands were separated using SDS-PAGE, and the proteins were then transferred to nitrocellulose membranes. The membranes were blocked using 5% bovine serum albumin (BSA) and subsequently incubated with 4HNE mouse mAb (#ABN249, Millipore Sigma) and GAPDH (G-9) Mouse mAb (#sc-365062) primary antibodies at a concentration of 1:1000 overnight in a cold refrigerator (4 °C). Depending on the sources of the primary antibodies, the membrane-bound antibodies were incubated with anti-rabbit/anti-mouse horseradish peroxidase (HRP)-coupled secondary antibodies (1:2000) for 1 h at room temperature. Immunolabeling was detected using ECL detection reagents (ThermoFisher Scientific) according to the manufacturer’s protocols. The images of the protein bands were taken with a FluorChem E imaging system. The intensity of the scanned WB images was analyzed with ImageJ software (NIH). The GAPDH protein was used as a loading control to normalize the proteins of interest.

### 2.9. Statistical Analysis

We compiled the experimental data and calculated the means and standard error of the means (SEM) using Excel spreadsheets. To determine the statistical significance between two groups, we performed the Student’s *t*-test, and for multiple groups, we employed one-way/two-way ANOVA, followed by Tukey’s post hoc test analysis, using GraphPad Prism 9.2.0.332 (GraphPad Software). We considered the level of significance at <0.05.

## 3. Results

### 3.1. PCB 126 Activates the Aryl Hydrocarbon Receptor and Increases Oxidative Stress in Cultured Mouse Coronary Endothelial Cells

**Figure 2:**
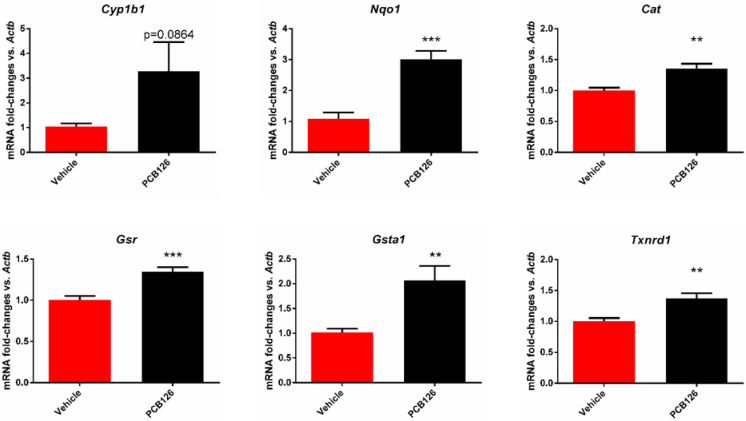
mRNA expression of *Cyp1b1, Nqo1*, *Cat*, *Gsr*, *Gsta1, and Txnrd1 in* PCB 126-treated MCECs. mRNA expression of AhR transcriptional target *Cyp1b1*, and the expression of oxidative stress-sensitive genes regulated by the NRF2 pathway, including *Nqo1*, *Cat*, *Gsr*, *Txnrd1*, and *Gsta1*, is measured in MCECs 24 h after being treated with the vehicle (Red) or 4.4 µm PCB 126 (Black). Sample size: *n* = 6. Data are shown as the mean ± SEM. ** *p* ≤ 0.01 and *** *p* ≤ 0.001; otherwise, the *p*-values are shown above the bar.

It is well-established that exposure to dioxin-like pollutants such as PCB 126 elicits a xenobiotic detoxifying response through the activation of the AhR [19,20]. To examine if the AhR targets were inducible in MCECs, we exposed the cells to 4.4 µM PCB 126 for 24 to 72 h. The PCB 126 treatment increased the expression of the AhR target gene *Cyp1b1* at 24 h (*p* = 0.086; Figure 2). A well-established mechanism of dioxin-like pollutant toxicity is an increase in oxidative stress. To examine this in the MCECs, we examined the mRNA expression of the genes known to be oxidative stress-sensitive. Here, we measured the representative antioxidant genes regulated by oxidative stress-sensitive NFE2-like BZIP Transcription Factor 2 (NFE2L2 or NRF2) [21] after vehicle or 4.4 uM PCB 126 treatment for 24 h. The expression of *Nqo1* (*p* = 0.0002), *Cat* (*p* = 0.0022), *Gsr* (*p* = 0.0009), *Gsta1* (*p* = 0.0064), and *Txnrd1* (*p* = 0.0031) were significantly increased due to PCB 126 treatment (Figure 2), and *Gpx2* and *Gstm1* showed no significant changes (data not provided).

**Figure 3:**
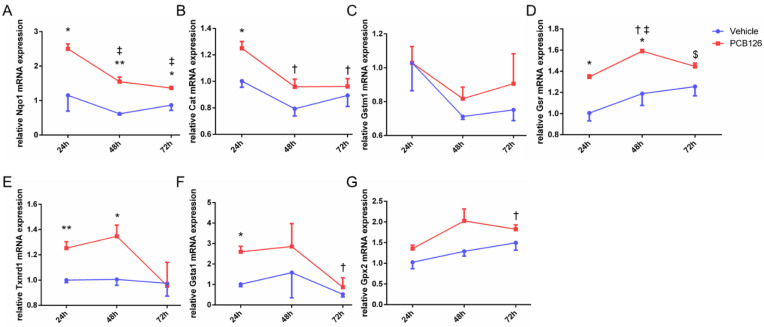
mRNA expression of 7 antioxidant genes, including *Nqo1* (**A**), *Cat* (**B**), *Gstm1* (**C**), *Gsr* (**D**), *Txnrd1* (**E**), *Gsta1* (**F**), and *Gpx2* (**G**), that are regulated by the NRF2 pathway is measured in the MCECs after 24, 48, and 72 h of treatment with the vehicle (Blue) or 4.4 um PCB 126 (Red). Sample size: *n* = 3. Data are shown as the mean ± SEM. When compared with the treatment counterparts, * *p* ≤ 0.05 and ** *p* ≤ 0.01. When compared with 24-h counterparts among the PCB 126-treated groups, † *p* ≤ 0.05, ‡ *p* ≤ 0.01, and † ‡ *p* ≤ 0.001. When compared with the 48-h counterparts among the PCB 126-treated groups, $ *p* ≤ 0.05.

To examine if the observed PCB 126-induced oxidative stress was resolved over time, we next completed a time course to include 48 and 72-h exposure durations. Our results were consistent with previous data that oxidative stress-sensitive genes were induced in the PCB-treated group (Figure 3). The expression of *Nqo1* showed a significant increase by PCB 126 treatment at all three time points: 24 h, 48 h, and 72 h, with an observed 2.2-fold (*p* = 0.0489), 2.5-fold (*p* = 0.0026), and 1.5-fold (*p* = 0.0351) increase compared with their vehicle counterparts, respectively (Figure 3A). Significant differences in the expression of catalase (Figure 3B) were observed 24 h after treatment (1.2-fold, *p* = 0.0228), but there was no significant difference between the vehicle or PCB 126 at 48 and 72 h. The expression of *Gsr* (Figure 3D) was significantly increased by the PCB 126 treatment at 24 h (1.3-fold, *p* = 0.0112) and 48 h (1.3-fold, *p* = 0.0240) but not at 72 h. Similarly, the expression of *Txnrd1* (Figure 3E) was significantly increased at 24 h (1.3-fold, *p* = 0.0096) and 48 h (1.3-fold, *p* = 0.0271), but a resolution occurred by 72 h. The expression of *Gsta1* (Figure 3F) was significantly increased due to PCB 126 only at 24 h (2.6-fold, *p* = 0.0058). The expression of *Gpx2* (Figure 3G) showed a similar trend to *Gsr* but did not reach significance at any of the three time points. Overall, most of the genes examined, except for *Gsr* and *Gpx2*, showed a downward trend of activation over time. For example, the expression of *Gsta1* (Figure 3F) in PCB 126-treated cells at 72 h was significantly decreased compared to at 24 h (0.3-fold, *p* = 0.0367).

### 3.2. PCB 126 Decreases ALDH2 Activity, and Pharmacological Inhibition of ALDH2 Exacerbates the PCB-Mediated Effects, Whereas the Pharmacological Activation of ALDH2 Rescues the PCB-Mediated Effects in Cultured Mouse Coronary Endothelial Cells

**Figure 4:**
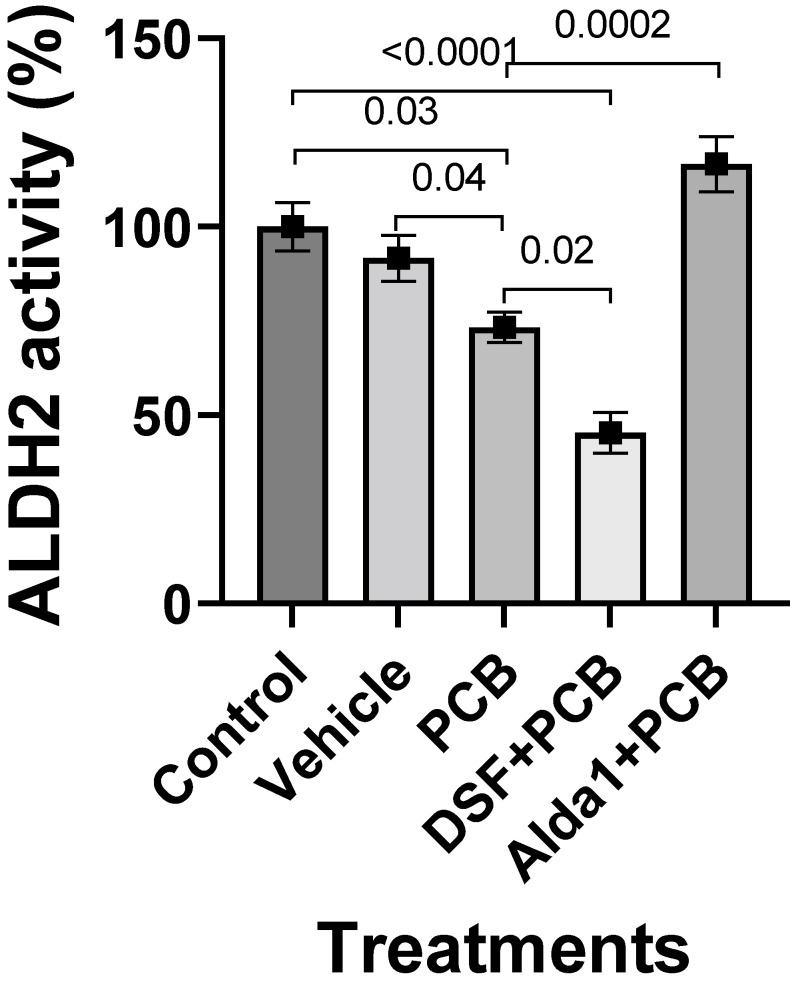
ALDH2 activity in PCB 126 and/or DSF/Alda 1-treated MCECs. Quantitative data of ALDH2 activity in MCECs after 24 h of PCB 126 treatment. *n* = 6 for each group. Each bar represents the mean ± SEM. DSF, disulfiram, PCB, polychlorinated biphenyl.

The 4.4 µM PCB 126-treated MCECs significantly decreased ALDH2 activity compared with the control (*p* = 0.03) (Figure 4). A 2.5 µM DSF pretreatment for 2 h, followed by the treatment with PCB 126 for 24 h, exacerbated the PCB 126-mediated decrease in ALDH2 activity (*p* = 0.02 vs. PCB 126 alone; *p* < 0.0001 vs. the control) (Figure 4). However, a 10 µM Alda 1 pretreatment for 2 h, followed by the treatment with PCB 126 for 24 h, rescued the PCB 126-mediated decrease in ALDH2 activity (*p* = 0.0002 vs. PCB 126 alone and *p* < 0.0001 vs. PCB 126 + DSF) (Figure 4).

### 3.3. PCB 126 Increases the 4HNE Protein Adduct Levels, Whereas the Pharmacological Activation of ALDH2 Rescues the PCB-Mediated Effects in Cultured Mouse Coronary Endothelial Cells

**Figure 5:**
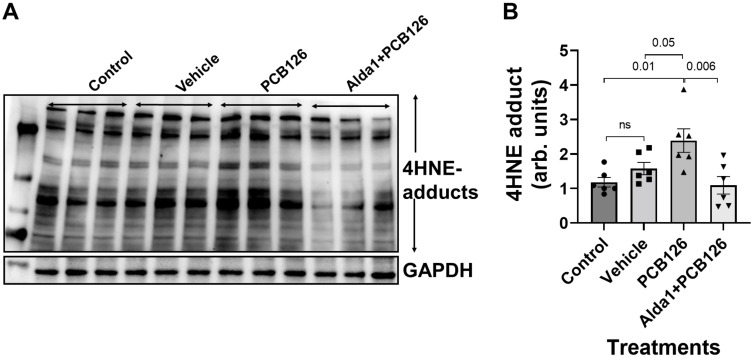
The 4HNE protein adduct levels in PCB 126 and/or Alda 1-treated MCECs. (**A**) Representative WB band image of the 4HNE protein adducts after 24 h of PCB 126 treatment. (**B**) Quantification of the WB data in (**B**). *n* = 6 for each group. Each bar represents the mean ± SEM. PCB, polychlorinated biphenyl; ns, not significant.

The 4.4 µM PCB 126 treatment in MCECs significantly increased the 4HNE protein adduct levels compared with the control (*p* = 0.01) (Figure 5). However, a 10 µM Alda 1 pretreatment for 2 h, followed by the treatment with PCB 126 for 24 h, rescued the PCB 126-mediated decrease in the 4HNE protein adduct levels (*p* = 0.006 vs. PCB 126) (Figure 5).

### 3.4. Pharmacological Inhibition of ALDH2 Exacerbates the PCB 126-Mediated Decrease in Angiogenesis, Whereas the Activation of ALDH2 Attenuates the PCB 126-Mediated Effect in Cultured Mouse Coronary Endothelial Cells

**Figure 6:**
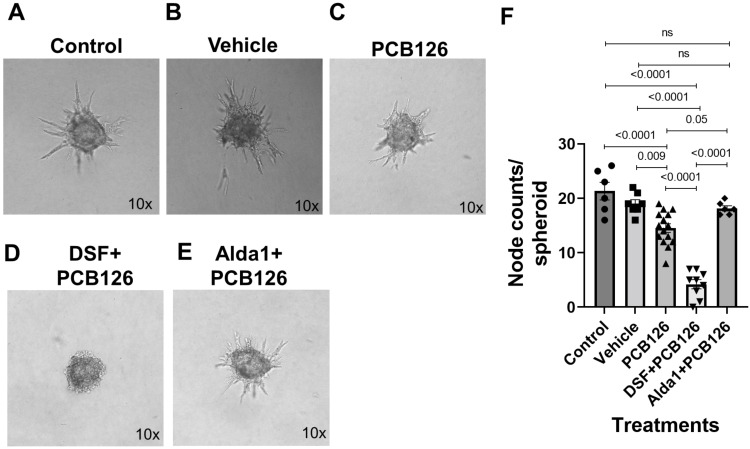
(**A**–**E**) Representative micrographs of sprout formation by the control, vehicle, 4.4 µM PCB126, 2.5 µM DSF + 4.4 µM PCB126, and 10 µM Alda1 + 4.4 µM PCB126-treated spheroids on a collagen matrix for 48 h, respectively. Magnification: 10×. (**F**) Quantitative data of the node counts/spheroids with different treatments stated in (**A**–**E**). *n* = 6–15 for each group. Each bar represents the mean ± SEM. DSF, disulfiram; PCB, polychlorinated biphenyl; ns, not significant.

The spheroid assay data showed that 4.4 µM PCB 126-treated MCECs significantly decreased sprout numbers compared with the control and vehicle (*p* < 0.0001 vs. the control and *p* = 0.009 vs. the vehicle) (Figure 6A–C,F). A 2.5 µM DSF pretreatment for 2 h, followed by the treatment with PCB 126 for 48 h, exacerbated the PCB 126-mediated decrease in the sprout counts (*p* < 0.0001 vs. both the control and PCB 126 alone) (Figure 6A,C,D,F). However, a 10 µM Alda 1 pretreatment for 2 h, followed by the treatment with PCB 126 for 48 h, rescued the PCB 126-mediated decrease in the sprout counts (*p* = 0.05 vs. PCB 126 alone and *p* < 0.0001 vs. PCB 126 + DSF) (Figure 6C–F).

**Figure 7:**
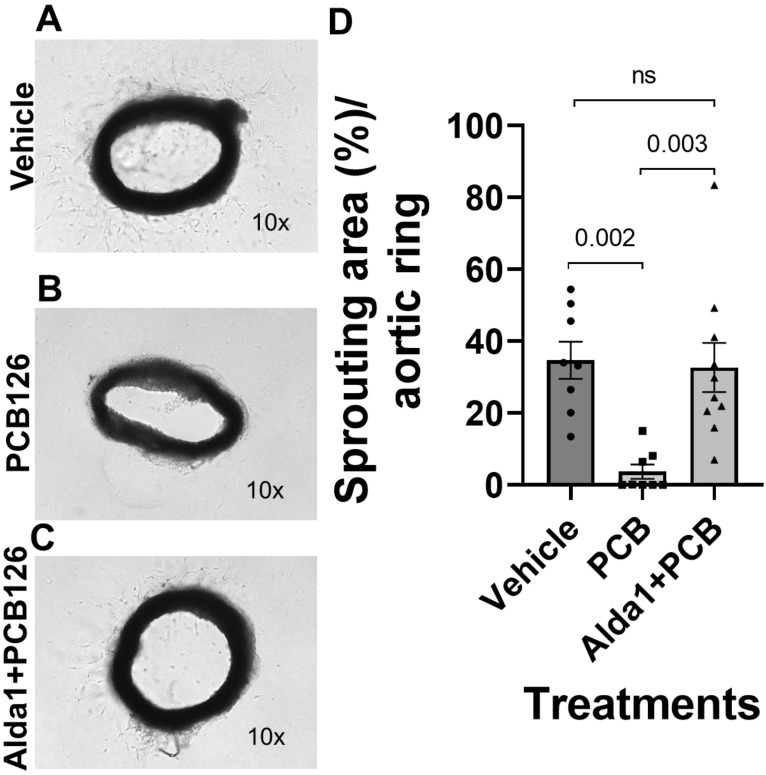
(**A**–**C**) Representative micrographs of sprout formation by the vehicle, 4.4 µM PCB126, and 10 µM Alda1 + 4.4 µM PCB126-treated aortic rings on the collagen matrix for 72 h, respectively. Magnification: 10×. (**D**) Quantitative data of the sprouting area/aortic ring with different treatments stated in (**A**–**C**). *n* = 8–10 for each group. Each bar represents the mean ± SEM. PCB, polychlorinated biphenyl; ns, not significant.

The aortic ring assay data showed that 4.4 µM PCB 126-treated aortic rings significantly decreased the sprouting area compared with the vehicle (*p* = 0.002) (Figure 7A,B,D). A 10 µM Alda 1 pretreatment for 2 h, followed by the treatment with PCB 126 for 72 h, rescued the PCB 126-mediated decrease in the sprout counts (*p* = 0.003 vs. PCB 126) (Figure 7B–D).

## 4. Discussion

This study suggests that PCB 126 increases AhR activation upregulation, as well as the expression of oxidative stress-sensitive genes and decreases angiogenesis in cultured MCECs by decreasing the activity of ALDH2 while increasing the 4HNE adduct levels. Some of these PCB 126-mediated effects were exacerbated by ALDH2 inhibition using DSF, whereas these effects were rescued by ALDH2 activation with Alda-1.

Dioxins and dioxin-like chemicals are a group of structurally related chemicals with long half-lives that are largely generated by humans through industrial processes, including incineration, the production of herbicides and pesticides, and the use of fertilizers [22]. Dioxins are highly toxic and have been found to work through a common mechanism of action that is mediated through the activation of AhR [23]. Similar to dioxins, man-made dioxin-like PCBs, such as PCB 126, have been manufactured and utilized as additives to sealants and paints and in oils used in industrial processes [24,25]. In this study, when we treated MCECs with PCB-126, there was a near-significant increase in the transcription of the *Cyp1b1* genes. Therefore, we can speculate on the activation of AhR and subsequent upregulation of *Cyp1b1* and other *AhR* target genes in MCECs.

These dioxin-like PCBs have a coplanar structure, leading to similar chemical characteristics, modes of action, and toxicities such as dioxins [26]. PCBs began to be mass produced in the early 1930s and have been well-studied as one of the most toxic classes of persistent organic pollutants of worldwide concern [24]. Due to their extreme chemical and thermal stability, PCBs are highly resistant to degradation, leading to bioaccumulation in the environment and in the fatty tissues of animals and humans [27]. As we mentioned earlier, PCBs were eventually banned in the United States in the late 1970s and then worldwide in 2001 after the Stockholm Convention [24,28]. Since this ban, the primary source of exposure to PCBs has been through contaminated food, including high-fat foods such as meat, fish, and dairy products [29,30]. Since PCBs are now ubiquitous contaminants in the global food chain, even humans in the general population have been exposed to PCBs [31]. Numerous epidemiological studies have found that exposure to PCBs is often associated with adverse human health effects, including vascular diseases (US EPA). Hypertension has been associated with increased serum levels of dioxin-like PCBs in highly exposed populations, especially in younger populations [32,33]. The data analysis from the NHANES (1999–2002) revealed that dioxin-like PCBs were positively associated with hypertension but only among men [34]. Dietary exposure to PCBs was also shown to be associated with increased coronary calcium and more intense atherosclerosis in the general male population [5]. Experimental evidence has further highlighted the relationship between exposure to dioxin-like PCBs and the accelerated development of atherosclerosis [13]. In 2013, a cross-sectional study of an elderly Swedish population revealed that high serum levels of PCBs were associated with dysfunction of the left ventricle, independent of other heart failure risk factors [35]. Furthermore, a study examining Swedish population-based prospective cohorts from 1997 to 2010 found that dietary PCB exposure was associated with an increased risk of HF in both women and men [8]. Thus, PCBs are associated with cardiovascular diseases. In our study, we tested whether PCB-126 can increase oxidative stress in cultured MCECs and found it enhanced the activation of AhR and corresponding antioxidant genes *Nqo1*, *Cat*, *Gsr*, *Gpx2*, *Gsta1*, and *Txnrd1*. For most of the markers of oxidative stress, the expression remained significantly elevated compared to the control even 72 h after exposure.

CMDs are one of the major leading causes of death worldwide. One of the contributing factors to CMDs is oxidative stress. Since the human heart is utilizing 15 to 20 times more than its own weight of the daily amount of adenosine triphosphate (ATP) for it to function [36], this puts the heart under huge oxidative stress. The lipid peroxidation of poly-unsaturated fatty acids (PUFAs) such as arachidonic acids by ROS and superoxide is a critical component of oxidative stress [37]. The most prominent products of lipid peroxidation are 4HNE-like reactive carbonyls [37]. Due to the oxidation process, the peroxyl radical addition and disintegration of cardiolipin on the mitochondrial lipid membrane generate considerable amounts of 4HNE [38]. 4HNE can form covalent adducts in several cellular signaling cascades [39]. Specialized enzyme systems such as glutathione S-transferases (GSTs), glutathione (GSH), aldose reductase (AR), and ALDH are involved in the detoxification of 4HNE-like reactive carbonyls.

We and others have shown that ALDH2 is key in conferring a cardioprotective role by detoxifying 4HNE and reducing 4HNE-mediated toxicity [16,17,37,40,41,42,43]. Diabetes-induced CEC dysfunction contributes to diabetic heart disease, another important type of CMD [15,44]. We reported that a decrease in ALDH2 activity, either by genetic modification [15] or pharmacological inhibition [1], exhibited poor angiogenesis. We also found that 4HNE is directly involved in this low ALDH2 activity-mediated decrease in coronary angiogenesis [15,18]. In another study, we show that ALDH2 activation improves Ang II-mediated defective CEC angiogenesis by decreasing 4HNE-mediated cytotoxicity [45]. Similarly, in the current study, we found that PCB-126 increased 4HNE and reduced ALDH2 activity, along with angiogenesis. We also found a pharmacological inhibition of ALDH2 with DSF-potentiated PCB-126-induced decreased coronary angiogenesis. However, the activation of ALDH2 by Alda-1 increased the coronary angiogenesis.

Thus, in conclusion, we propose that ALDH2 can be an important target in reducing PCBs such as pollutant-mediated cellular toxicity in endothelial cells. Future studies utilizing human cardiac endothelial cells and well-established rodent models of heart failure will help to establish a clearer directive relationship between dioxin-like pollutant exposure and heart failure risk. Finally, we conclude that ALDH2 activation can be a therapeutic strategy to improve coronary angiogenesis to ameliorate CMDs like HFpEF.

## Figures and Tables

**Table 1:** List of primers used for qPCR.

Gene	Forward	Reverse
NQO1	GGCATCCAGTCCTCCATCAA	GTTAGTCCCTCGGCCATTGTT
CAT	CAGAGAGCGGATTCCTGAGAGA	CTTTGCCTTGGAGTATGTGGTGAT
GSTM1	ATACTGGGATACTGGAACGTCC	AGTCAGGGTTGTAACAGAGCAT
GSR	TCGGAATTCATGCACGATCA	GGCTCACATAGGCATCCCTTT
TXNRD1	GGCCAAAATCGGTGAACACATGGAAG	CGCCAGCAACACTGTGTTAAATTCGCCC
GSTA1	AAGCCCGTGCTTCACTACTTC	GGGCACTTGGTCAAACATCAAA
GPX2	GTGGCGTCACTCTGAGGAACA	CAGTTCTCCTGATGTCCGAACTG
CYP1B1	AATGAGGAGTTCGGGCGCACA	GGCGTGTGGAATGGTGACAGG
ACTB	GCCACTGTCGAGTCGCGT	GATACCTCTCTTGCTCTGGGC

## Data Availability

All relevant data are within the paper. All data are fully available without restrictions.
